# Exosomes Promote Pre-Metastatic Niche Formation in Gastric Cancer

**DOI:** 10.3389/fonc.2021.652378

**Published:** 2021-05-24

**Authors:** Jing Gao, Song Li, Qian Xu, Xue Zhang, Miao Huang, Xin Dai, Lian Liu

**Affiliations:** ^1^ Department of Medical Oncology, Qilu Hospital, Cheeloo College of Medicine, Shandong University, Jinan, China; ^2^ Department of Oncology, Affiliated Hospital of Shandong University of Traditional Chinese Medicine, Jinan, China

**Keywords:** exosome, gastric cancer, pre-metastatic niche, metastasis, tumor microenvironment

## Abstract

Gastric cancer has a high rate of metastasis, during which pre-metastatic niches (PMN) provide a supportive environment for the upcoming tumor cells. Exosomes are bilayer vesicles secreted by cells containing biological information that mediates communication between cells. Using exosomes, gastric cancer cells establish PMN remotely in multifarious perspectives, including immunosuppression, stroma remodeling, angiogenesis, mesothelial mesenchymal transformation, and organotropism. In turn, the cell components in PMN secrete exosomes that interact with each other and provide onco-promoting signals. In this review, we highlight the role of exosomes in PMN formation in gastric cancer and discuss their potential values in gastric cancer metastasis diagnosis, prevention, and treatment.

## Introduction

Gastric cancer (GC) is one of the most common cancers globally, ranking the fifth in cancer incidence and the third in cancer-related death (1). Despite that surgery and perioperative radiotherapy or chemotherapy are the primary treatments for early-stage gastric cancer, more than half of patients with radical resection suffered local recurrence or distant metastasis (2). Moreover, many patients were initially diagnosed with metastatic gastric cancer that is unresectable (3). Due to high heterogeneity and drug resistance, the median survival rate of metastatic gastric cancer rarely exceeds one year, and the 5-year survival rate is less than 10% (4). In particular, peritoneal metastasis is the most common metastatic pattern of gastric cancer and has several negative features, including high incidence, high mortality, difficult diagnosis, and poor prognosis (5). It occurs in 55%-60% of advanced gastric cancers (6) and has a 5-year survival rate of only 2% (7).

One of the crucial steps during metastases is the formation of a pre-metastatic niche (PMN), which provides a receptive and supportive environment in terms of nutrients, extracellular matrix (ECM), stromal cell, and immune cells for cancer cells to seed in distant organs (8–11). The PMN is initiated and educated by PMN-promoting molecules secreted by the primary tumors, tumor-mobilized myeloid cells, and local stromal cells of the host. These niche-promoting molecules include tumor-secreted factors, cytokines, chemokines, inflammatory factors, microvesicles, oncosomes, and exosomes (11).

Recently, studies revealed that exosomes play essential roles in PMN formation (12). Ranging from 40 to 150 nm in size and enveloped by lipid bilayers, exosomes are a class of extracellular vesicles released by various cells (13). They contain a variety of cell components, including DNA, RNA, lipids, and proteins, that can be transported to and can regulate recipient cells (13). Importantly, they carry a variety of cytokines, such as TGF-β, TNF-α, IL-6, IL-8, and IL-10, to maintain their stability and transfer to distant recipient cells (14). In particular, tumor-derived exosomes (TDEs) travel from their original site to distant potential metastatic sites and educate the PMN components with their cargo, thereby facilitating tumor cell arrival and colonization (15). In addition, exosomes from non-tumor cells may also participate in this process (15–18). In this review, we summarize the roles of exosomes in PMN formation in gastric cancer, as well as their implications in gastric cancer metastasis, prevention, and treatment.

## Roles of Exosomes in Gastric Cancer Malignity

**Figure 1:**
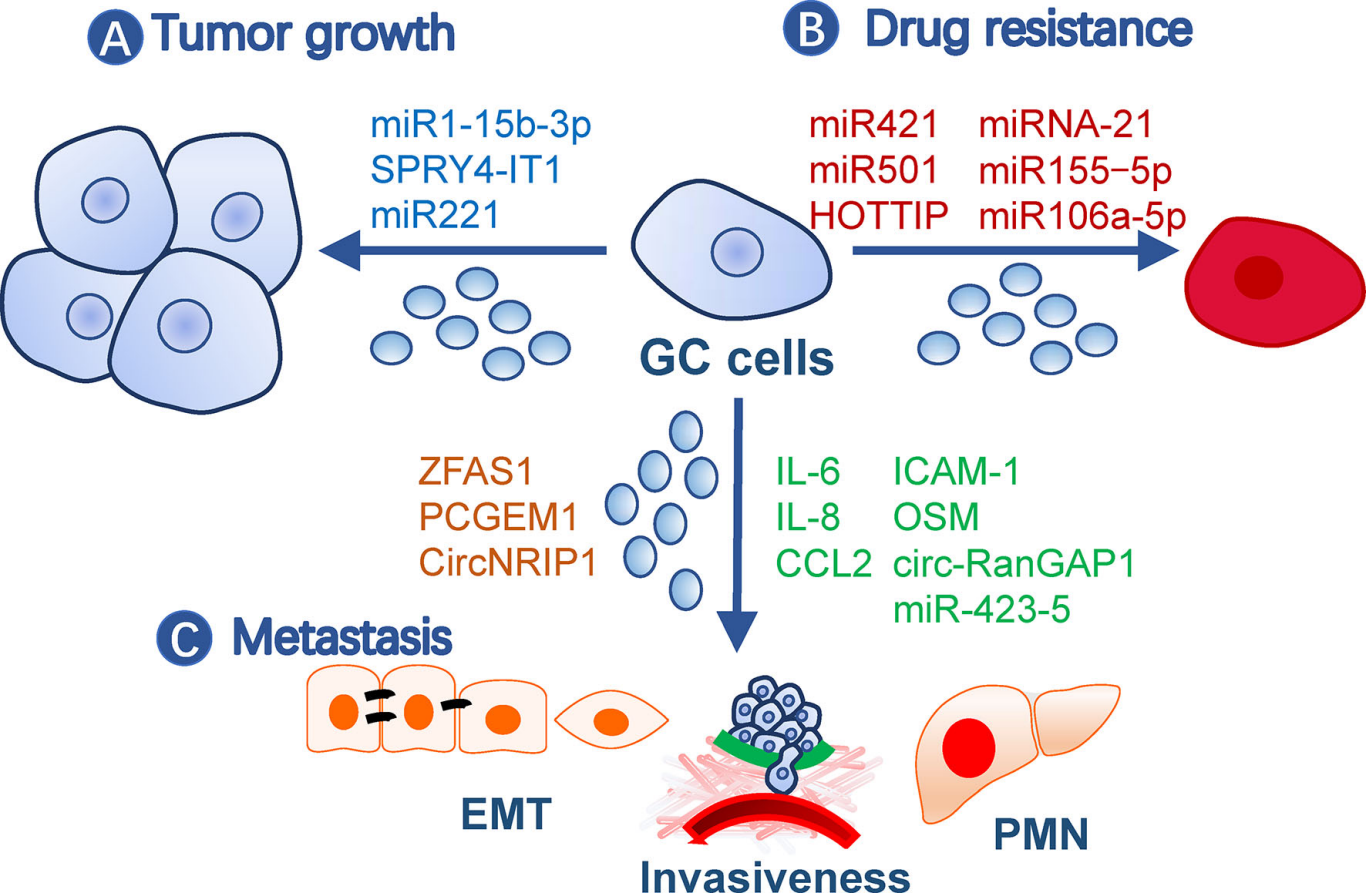
Roles of exosomes in gastric cancer malignity. Exosomes from gastric cancer cells or other cells contribute to malignity in **(A)** tumor growth, **(B)** drug resistance, and **(C)** metastasis. Exosomes may affect all processes of metastasis, such as EMT, invasiveness, and pre-metastatic niche formation. The small circles represent exosomes, and effective molecules were listed by the arrows. EMT, Epithelial-mesenchymal transition; GC, gastric cancer; PMN, pre-metastatic niche.

Exosomes contribute to malignity through their roles in gastric cancer growth, drug resistance, and metastasis (**Figure 1**).

### Promotion of Gastric Cancer Growth

Gastric cancer TDE contents, including proteins and nucleic acids, have a broad impact on tumor growth. For example, TDEs secreted by BGC-823 cells can transfer miR-15b-3p to SGC-7901 or GES-1 cells to enhance their development by inhibiting the DYNLT1/Caspase-3/Caspase-9 signaling pathway (19). SPRY4-IT1, a long noncoding RNA (lncRNA), promotes gastric cancer proliferation and migration by sponging miR-101-3p, is upregulated in serum exosomes of gastric cancer patients, and is correlated with patient outcomes (20). In addition, exosomes from non-tumor cells may also impact tumor growth. For example, exosomes from gastric cancer tissue-derived mesenchymal stem cells (MSCs) are onco-promoting by transmitting miR-221 (21), while gastric mucosal epithelial cells induce apoptosis of gastric cancer cells through exosomal proteins (22).

### Induction of Drug Resistance

Gastric cancer cells may transmit drug resistance to other sensitive clones by communication with exosomes. For instance, doxorubicin-resistant SGC7901 cells conferred the same drug resistance in drug-sensitive cells *via* exosomal miR-501 that targets BLID (23). Cisplatin resistance was transmitted by exosomal lncRNA HOTTIP, which targets the miR-218/HMGA1 axis in cisplatin-sensitive cells (24). M2 macrophage-derived exosomes also transferred cisplatin-resistance through miRNA-21 targeting PETN in recipient cells (25). A paclitaxel-resistant gastric cancer cell line, MGC803R, can induce chemoresistance in paclitaxel−sensitive cells, MGC803S, by exosomal delivery of miR−155−5p, which further suppresses GATA3 and TP53INP1 in the latter (26). In addition, TFAP2E hypermethylation facilitates packaging of miR-106a-5p and miR-421 into gastric cancer exosomes, which subsequently induce 5−fluorouracil resistance in tumor cells (27).

### Facilitation of Gastric Cancer Metastasis

Metastasis is a multistep process including cancer cell motility, local infiltration, intravasation, transit in the blood or lymph, extravasation, and proliferation in competent organs (28). Epithelial-mesenchymal transition (EMT) is a biological process associated with increased cell motility, resistance to apoptosis and senescence, and suppressed immune reaction during the initial step of metastasis (29) and is closely regulated by TDEs (30). LncRNA ZFAS1 and PCGEM1 are highly enriched in gastric cancer TDEs and are capable of inducing EMT phenotypes among cancer cells during metastasis, in which PCGEM1 stabilizes SNAI1 (31, 32). CircNRIP1, a circular RNA, can also be transmitted among gastric cancer cells through exosomes and regulate EMT through a circNRIP1-miR-149-5p-AKT1/mTOR axis (33). In addition, exosomal TRIM3 was found to be an anti-EMT factor, and its levels were downregulated in gastric cancer TDEs (34). Besides TDEs, malignant ascites-derived exosomes also played essential roles in enhancing the EMT signaling in gastric cancer cells during peritoneal metastasis (35). Invasiveness is also critical during the initial and terminal processes of tumor metastasis and is easy to be quantified by *in vitro* and *in vivo* methods. This ability of gastric cancer cells can be elevated by exosomal miR-423-5p, which is remarkably correlated with lymph node metastasis (36). In SGC cells, CD97 facilitated cell invasions through packaging miRNAs into exosomes, which mediated the MAPK signaling pathway (37). circ-RanGAP1 is elevated in plasma exosomes from gastric cancer patients and facilitates gastric cancer invasiveness by upregulating VEGFA expression (38). Besides, omentum may play an active role in enhancing gastric cancer cell invasiveness by exosomal proteins (i.e., IL-6, IL-8, ICAM-1, CCl2, and OSM) (39).

Besides these processes, PMN formation is also indispensable for distant metastasis and is closely regulated by exosomes. Next, we will focus on the roles of exosomes in gastric cancer PMN formation.

## Roles of Exosomes in Gastric Cancer PMN Formation

**Figure 2:**
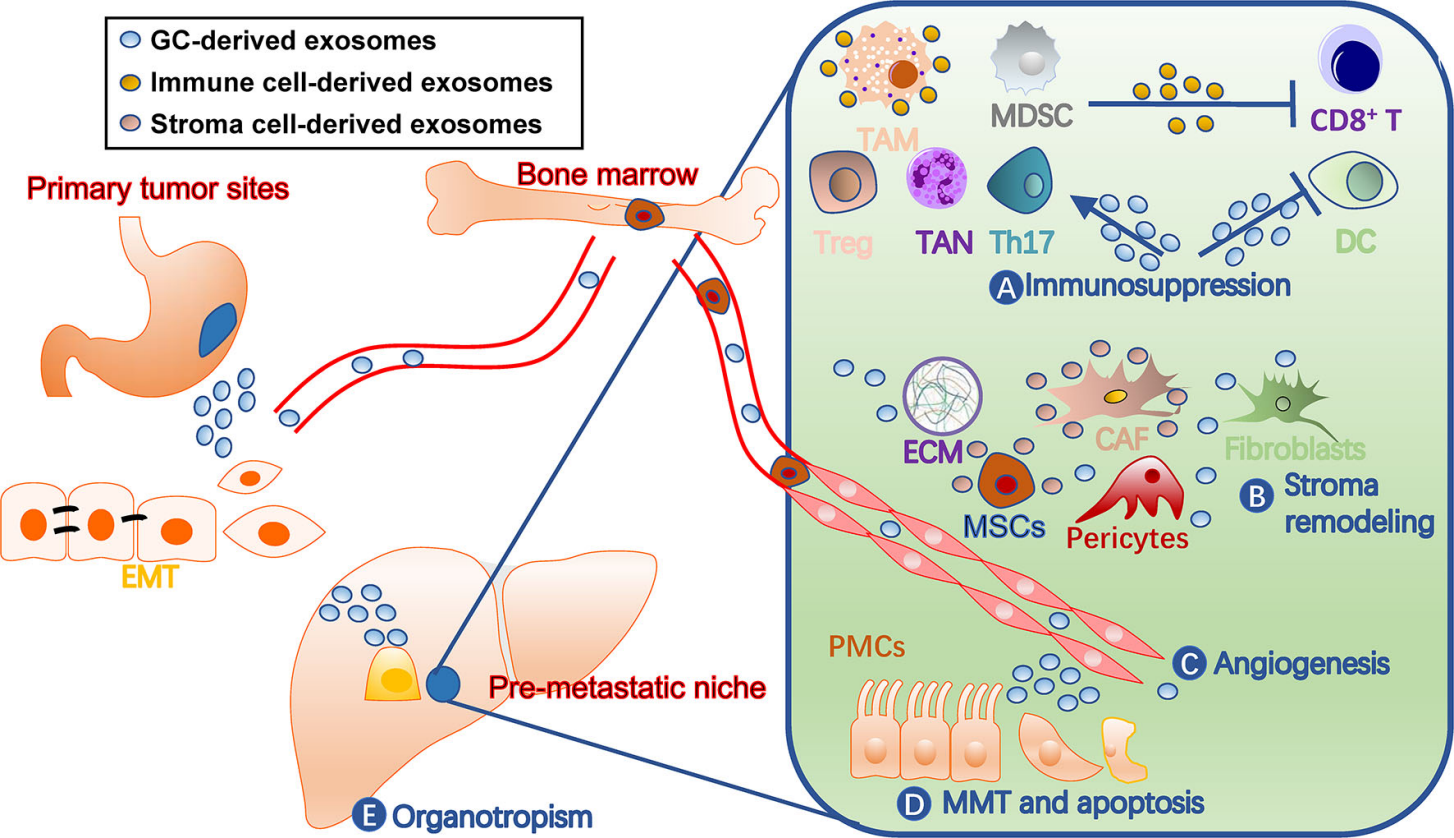
Roles of exosomes in gastric cancer pre-metastatic niches. Exosomes contribute to pre-metastatic niche formation through multiple mechanisms, including **(A)** immunosuppression by facilitating TAM and TAN polarization, inhibiting dendritic cell maturation and T cell activation, and inducing MDSCs, **(B)** stroma remodeling by acting on stromal cells such as CAF and MSCs and ECM balance, **(C)** angiogenesis, **(D)** MMT and apoptosis of tissue-specific cells, such as PMCs, and **(E)** organotropisms. The liver is drawn to represent a metastatic target. CAF, cancer-associated fibroblast; DC, dendritic cell; ECM, extracellular matrix; EMT, epithelial-mesenchymal transition; GC, gastric cancer; MDSCs, myeloid-derived suppressor cells; MMT, mesothelial-mesenchymal transition; MSC, mesenchymal stem cell; PMC, peritoneal mesothelial cell; TAM, tumor-associated macrophage; TAN, tumor-associated neutrophil; Th17, T-helper 17 cell; Treg, regulatory T cell.

A PMN has distinct characters from normal tissue environments, such as immunosuppression, angiogenesis, and organotropism (11). Cells involved in shaping these features are fine-tuned by tumor-secreted factors, including TDEs (**Figure 2**). They not only act on various immune cells for immunosuppression, but also remodel stromal components into tumor-supporting types in PMN. Additionally, exosomes target endothelial cells for angiogenesis and organ-specific cells, such as peritoneal mesothelial cells (PMCs), for organotropism. In turn, the immune and stromal cells secrete exosomes that benefit tumor cells and communicate among different cells (40).

### Immunosuppression

**Figure 3:**
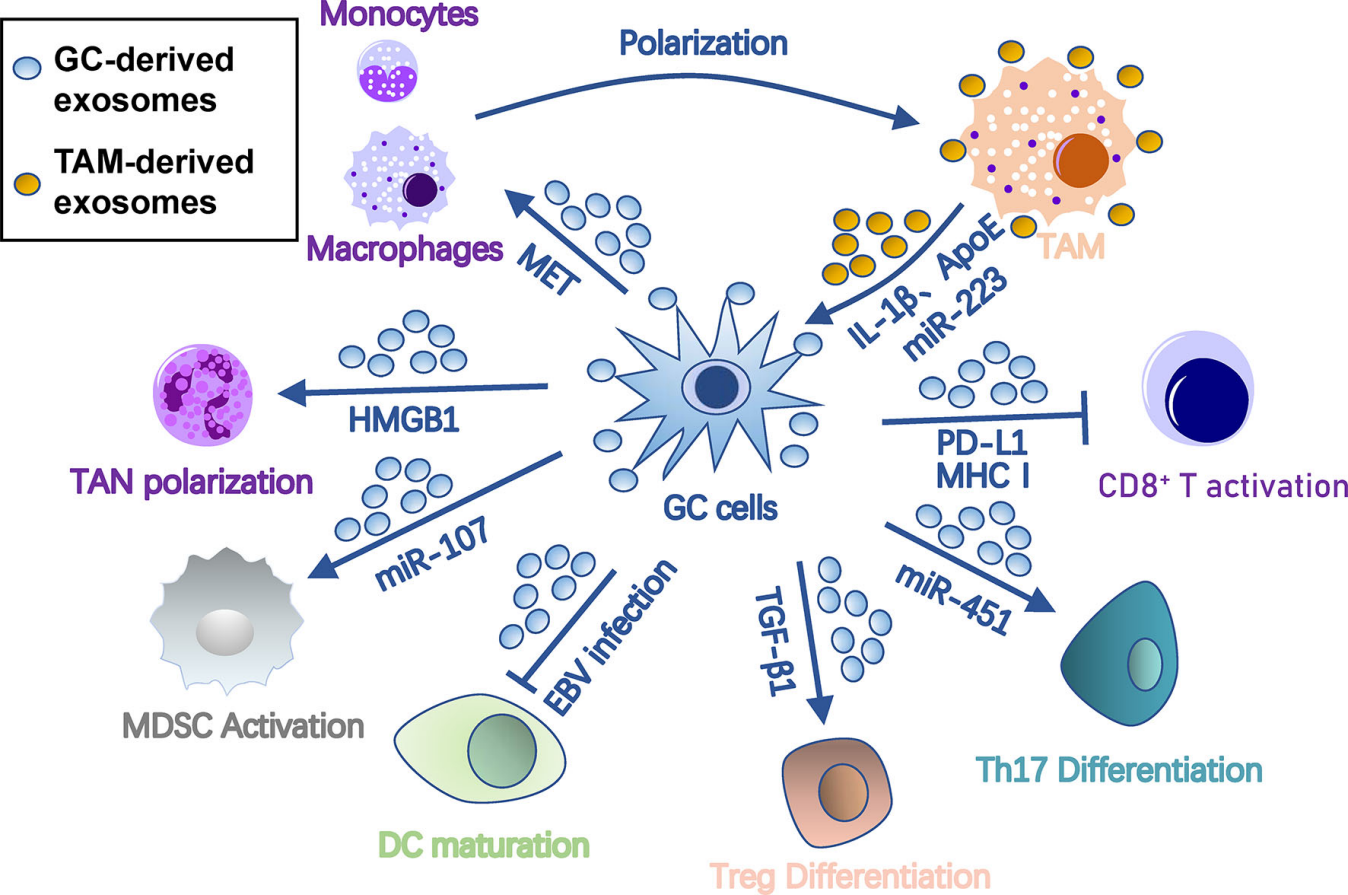
Roles of exosomes in immunosuppression in gastric cancer pre-metastatic niches. Gastric cancer-derived exosomes cause immunosuppression by suppressing DC/CD8^+^ T cell activation and DC maturation and inducing differentiation of TAM, TAN, MDSC, Treg, and Th17 cells. In turn, TAM provides onco-promoting signals to tumors and stromal cells through its exosomes. Arrows represent activation, whereas bar-headed arrows represent inhibition. CAF, cancer-associated fibroblast; DC, dendritic cells; ECM, extracellular matrix; EMT, epithelial-mesenchymal transition; MDSC, myeloid-derived suppressor cell; MMT, mesothelial-mesenchymal transition; MSC, mesenchymal stem cell; PMC, peritoneal mesothelial cell; TAM, tumor-associated macrophage; TAN, tumor-associated neutrophil; Th17, T-helper 17 cell; Treg, regulatory T cell.

To metastasize, tumor cells must evade immune surveillance and killing in the seeding organ. Thus, the PMN must orchestrate immunosuppression of all types of immune cells to protect colonizing tumor cells from immune attack. Exosomes are one of key factors that orchestrate this process among cancer cells, PMN, and immune cells (**Figure 3**). Specifically, exosomes polarize tumor-associated macrophages (TAMs), induce tumor-associated neutrophils (TANs), inhibit dendritic cell maturation, regulate T cell differentiation and function, and induce myeloid-derived suppressor cells (MDSCs).

#### Polarization of TAMs

Macrophages are the most abundant immune cells with two types of polarized cells, the tumor-suppressing M1 and tumor-promoting M2 (41). TAMs are primarily M2 polarized, capable of promoting tumors by regulating tumor malignancy, angiogenesis, and anti-tumor immunity (42). In gastric cancer, TDEs promote M2 transformation and facilitate production of pro-inflammatory factors by stimulating NF-κB pathways in macrophages (43). Gastric cancer TDEs also induce monocytes to differentiate into PD-1+ macrophages, which exhibit an M2-like surface profile and impair CD8+ T cell function (44). In Helicobacter pylori-infected gastric cancer, TDEs are enriched with activated MET, which educates the macrophages towards an M2 phenotype with high IL-1β expression (45).

In turn, TAM-derived exosomes may act on gastric cancer cells. ApoE is a highly specific protein in TAM-derived exosomes that can activate the PI3K-Akt signaling pathway in gastric cancer cells to rebuild the cytoskeleton for migration (46). Exosomal miR-223 derived from macrophages can provide proliferation/EMT signals for metastasis via the PTEN-PI3K/AKT pathway (47). Interestingly, during peritoneal PMN formation, TAM can transfer TDEs from gastric cancer cells to surrounding stromal cells, including peritoneal mesothelial cells, fibroblasts, and endothelial cells, and induce their conversion into cancer-associated fibroblasts (CAF)-like cells (48).

#### Induction of TANs

Like macrophages, neutrophils can also polarize into N1 or N2 phenotypes. N1 cells possess anti-tumor activity due to their immune-activating cytokines/chemokines that recruit and activate CD8+ T cells (49), while most TANs appear to have “pro-tumorigenic” N2 phenotypes (50). TANs release multifarious pro-PMN substrates, including reactive oxygen species and reactive nitrogen species that cause genetic instability and carcinogenesis (51), elastase that degrades tumor-suppressing proteins (52), prostaglandin E2 that drives wound inflammation-mediated pre-neoplastic cell proliferation (53), VEGF and MMP9 that facilitate angiogenesis (54), and CCL2/17 that recruits other immunosuppression cells (55, 56). In gastric cancer, membrane HMGB1 on TDEs interacts with TLR4 on neutrophils, which induces TAN polarization through NF-κB and an autophagic response to reshape the metastatic niche (57).

#### Inhibition of Dendritic Cell Maturation

Dendritic cells are the central antigen-presenting cells in anti-tumor immunity. Epstein-Barr virus (EBV)-positive gastric cancer is characterized by an abundance of infiltrated-immune cells, including dendritic cells, but with suppressed anti-tumor immunity. Hinata et al. revealed that EBV-positive gastric cancer has more TDE secretion than EBV-negative gastric cancer (58). These TDEs suppress dendritic cell maturation, represented with low CD86 expression, and inhibit activation of other immune cells (58).

#### Regulation of T Cell Differentiation and Function

Anti-tumor immunity is largely imposed by T cells, which are potential targets of TDEs for immunosuppression. Gastric cancer TDEs can induce T cell apoptosis by mediating PI3K proteasome degradation and caspases 3, 8, and 9 activation (59). TDE-bound PD-L1 is an immune response “brake” that is more stable than soluble PD-L1 and is co-expressed with MHC-I. Thus, TDE-bound PD-L1 is able to lead to much stronger T cell dysfunction than soluble PD-L1 in gastric cancer (60).

A group of immunomodulatory T cells, such as regulatory T (Treg) cells, are essential in coordinating distinct immunoregulatory programs and creating an immunologically permissive environment for tumor metastasis (61). Gastric cancer TDEs also induce FOXP3+ Treg cell differentiation from naive T cells through exosomal TGF-β1 (62). In addition, glucose deprivation can induce an exosome-mediated miR-451 redistribution from tumor to T cells, resulting in T-helper 17 cell differentiation in gastric cancer (63). A lncRNA, RP11-323N12.5, is found upregulated in gastric cancer cells as well as tumor-infiltrated lymphocytes (64). Upregulated RP11-323N12.5 in tumor-infiltrated lymphocytes is derived from TDEs and contributes to Treg differentiation by upregulating the Hippo pathway effector, YAP1 (64).

In gastric cancer, TDEs may also regulate T cell functions indirectly by a third cell. They can regulate immunomodulation function of MSCs through the NF-κB signaling pathway, which further activates CD69 and CD25 on the surface of T cells (65). Furthermore, gastric cancer TDEs also have immunogenicity to induce dendritic cell maturation for tumor-specific T cell responses (66).

#### Recruitment of MDSCs

MDSCs are a heterogeneous population of immature myeloid cells that suppress immunity during tumor progression, inflammation, or infection (67). They are indispensable for PMN formation due to their roles in immunosuppression, vascular permeability, and collagen remodeling (68). Many immune cells in PMNs, including cytotoxic T cells, Treg cells, NKT cells, dendritic cells, and macrophages, are regulated by MDSCs during tumor metastasis (69). TDEs have been shown to transform bone marrow myeloid cells into MDSCs (70). In gastric cancer, TDEs also help MDSC expansion by delivering miRNA-107 targeting DICER1 and PTEN-PI3K signaling in the recipient MDSC (71).

### Stroma Remodeling

**Figure 4:**
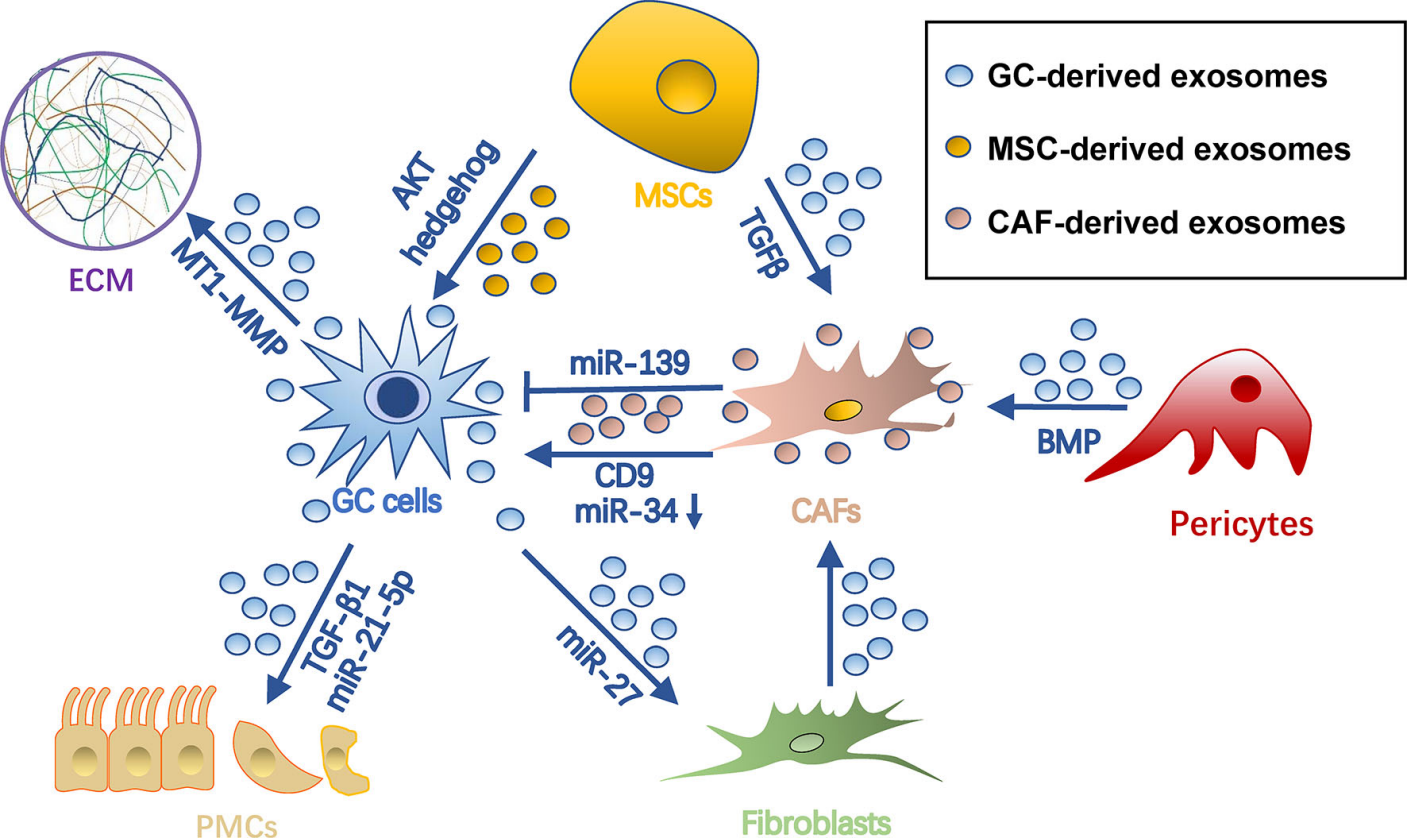
Roles of exosomes in stroma remodeling in gastric cancer pre-metastatic niches. Gastric cancer cell-derived exosomes can convert pericytes, fibroblasts, and MSCs into CAFs. Also, they remodel ECM and induce MMT and apoptosis of PMCs through different cargo during PMN formation. Meanwhile, exosomes from MSCs and CAFs can in turn promote tumor cell progression. Arrows represent activation, whereas bar-headed arrows represent inhibition. CAF, cancer-associated fibroblast; ECM extracellular matrix; MSC, mesenchymal stem cell; PMN, pre-metastatic niche.

The survival of cancer cells in the metastatic site highly depends on the stromal microenvironment, which is composed of fibroblasts, pericytes, MSC, endothelial cells, ECM, and vasculature (72). TDEs have been shown to remodel stroma by reprograming these stromal cells and ECMs for tumor colonization (**Figure 4**).

#### Regulation of CAF Reprogramming

CAFs are a group of heterogeneous cells that may differentiate from multiple origins (73) and are potent regulators of tumors and tumor microenvironments (74, 75). Exosomes are important for inducing pro-tumorigenic/metastatic CAFs. For example, gastric cancer TDEs contain high levels of miRNA-27a and can deliver them into fibroblasts for CAF reprogramming in a CSRP2-dependent manner (76). Conversely, CAFs with miRNA-27a provide a favorable environment pleiotropically for malignant behavior of gastric cancer cells (76). TGF-β on gastric cancer TDEs triggers differentiation of umbilical cord-derived MSC into CAF by activating the Smad pathway (77). In addition, gastric cancer TDEs induce transition of pericytes into CAFs by exosome-mediated BMP transfer and PI3K/AKT and MEK/ERK pathway activation (78).

Exosomes from CAF in turn impact gastric cancer cells. CAF-derived CD9+ exosomes potently stimulate MMP2 expression and migration in scirrhous-type gastric cancer cells (OCUM-12 and NUGC-3 cells) (79). This process was hindered by adding CD9 neutralizing antibodies or siRNAs targeting CD9 (79). In addition, CAF-derived exosomes are internalized by gastric cancer cells and facilitate gastric cancer proliferation and invasion (80). This process was mediated by exosomal miRNA-34, which targets 16 onco-promoting molecules in gastric cancer (80). Notably, not all CAF-derived exosomes are onco-promoting. For instance, the exosomal miR-139 derived from gastric cancer CAFs is anti-metastatic, decreasing MMP11 in tumor microenvironments (81).

#### Onco-Promoting Exosomes Derived from MSCs

MSCs are another origin of tumor stromal cells, which are able to differentiate into several types of mesenchymal cells, including adipocytes, CAFs, pericytes, and endothelial-like cells (82). They orchestrate an environment associated with tumor survival, angiogenesis, and immunosuppression, which all contribute to tumor growth and metastasis (83). MSC-derived exosomes have been proven indispensable during this process. In GC, they promote tumor growth and migrations *via* activation of the AKT (84) or hedgehog pathway (85). Besides, bone marrow-derived MSC exosomes are able to activate the ERK1/2 pathway in gastric cancer cells to upregulate VEGF expression, promoting angiogenesis (86). In a gastric cancer precancerous model, p53 deficient bone marrow-MSCs could secrete UBR2-rich exosomes, which activate the Wnt/catenin pathway in tumor cells and result in tumor growth, motility, and stemness (87). MSC exosomes could also induce chemoresistance in gastric cancer cells by antagonizing 5-fluorouracil-induced apoptosis and enhancing expression of multidrug resistance-associated proteins (88). This process is mediated by the calcium/calmodulin-dependent protein kinases and Raf/MEK/ERK kinase cascades (88).

#### Alternation of ECM Dynamics

ECM is a dynamic extracellular environment with continuous degradation, deposition, and modification and functions to fine-tune the elasticity and compressive or tensile strength of tissues (89). Dysregulated ECM remodeling includes irreversible proteolysis and crosslinking, which in turn influences microenvironmental cues, angiogenesis, and tissue biomechanics, being crucial for PMN formation (90). As far as we know, exosomes not only influence ECM dynamics directly, but also induce imbalances of metalloproteinases (MMPs) and tissue inhibitors of MMPs (TIMPs) released by tumor or stromal cells. Being rich in stable MMPs, TDEs help degrade collagen, laminin, and fibronectin to reshape ECM (91, 92). mRNA of membrane type 1-MMP is contained in and protected by gastric cancer TDEs and is highly associated with lymphatic metastasis (93). CD63 is an exosome marker as well as a receptor for TIMP1 (94). CD63 positivity in gastric cancer cells or gastric cancer stromal cells is significantly correlated with lymph node metastasis, so it is inferred that CD63-positive exosomes of gastric cancer might also be associated with metastatic niche formation (95), probably by binding with and inhibiting TIMP1. CD9+ exosomes derived from CAF also potently stimulate MMP expression in tumor environments (79).

### Inducing PMC Mesothelial-Mesenchymal Transition

The peritoneum is composed of a layer of PMCs and connective tissues and is the first barrier to tumor attachment and invasion in peritoneal metastasis (96, 97). Mesothelial-mesenchymal transition (MMT) has been observed to occur early in intraperitoneal dissemination, thus being an important process of peritoneal PMN formation (98, 99). Through MMT, mesothelial cells acquire a migratory phenotype expressing pro-inflammatory cytokines, angiogenetic factors, and a specialized ECM to disintegrate the peritoneum (100). Gastric cancer TDEs are able to induce MMT of PMCs (35, 101–103). For example, TDE-derived miR-21-5p can promote MMT of PMCs by suppressing SMAD7 (101). In addition, internalization of gastric cancer TDEs upregulates expression of adhesion-related molecules, including fibronectin-1 and laminin-γ1, in PMCs and promotes PMC-gastric cancer cell adhesion, which favors tumor settlement (102). Additionally, gastric cancer TDEs also elicit mesothelial barrier disruption and fibrosis by inducing concurrent apoptosis and MMT (103). Ascites-derived exosomes also potently facilitated TGF-β1-induced MMT of PMCs during gastric cancer peritoneal metastasis (104).

### Angiogenesis

**Figure 5:**
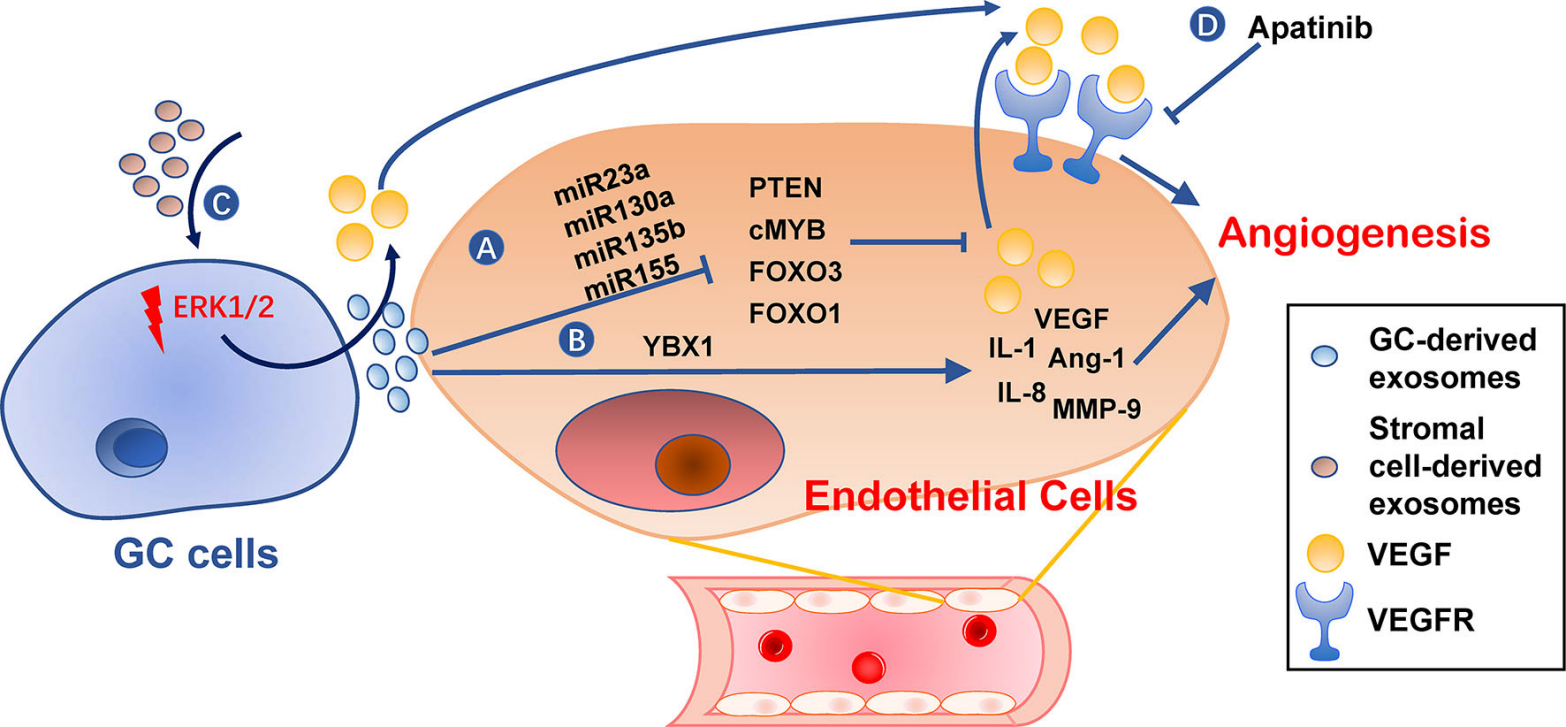
Roles of exosomes in angiogenesis. **(A, B)** Gastric cancer cell-derived exosomes can induce expression of pro-angiogenesis molecules, such as VEGF, IL-1, IL-8, Ang-1, and MMP-9, in endothelial cells by delivering miRNAs **(A)** or YBX1 **(B)**. **(C)** Stromal cell-derived exosomes or tumor-derived exosomes also promote VEGF expression in gastric cancer cells. **(D)** VEGFR inhibitors, such as Apatinib, can hinder angiogenesis caused by these exosomes. GC, gastric cancer.

Angiogenesis is an essential step in the establishment of PMN by providing oxygen and nutrients for tumor growth and allowing circulating tumor cells to arrive (105). Furthermore, angiogenic factor VEGF has an immunosuppressive effect during PMN establishment (106, 107). Gastric cancer TDEs can activate angiogenesis by delivering several types of miRNAs to vascular endothelial cells, including miR-130a targeting c-MYB (108), miR-135b targeting FOXO1 (109), miR-155 targeting FOXO3 (110) and c-MYB (111), miR-23a targeting PTEN (112) (**Figure 5**). Transmembrane protein Tetraspanin 8 on gastric cancer TDEs can also activate vascular endothelial cells by stimulating its ERK/MAPK pathway (113). Exosomal YB-1 from gastric cancer also promotes angiogenesis from endothelial cells by upregulating specific angiogenic factors (114). Irradiation-treated gastric cancer can produce TDEs with enhanced ability to induce angiogenesis of vascular endothelial cells (115). Consequently, VEGFR inhibitor, Apatinib, inhibits this process and could be used in combination with radiotherapy for better results (115). In addition, exosomes derived from non-tumor cells, such as MSCs, may also be angiogenic (86).

### Organotropism

Metastatic organotropism is one of the characteristics of PMN (11). Organotropism results from an active selection and education by the primary gastric cancer cells of a specific distant microenvironment into a PMN (116). As reported by Hoshino and colleagues, integrin patterns in TDEs determine the organotropic metastases through an integrin-dependent uptake of exosomes by different organs (117). For example, exosomal integrins α6β4 and α6β1 are lung-tropic, while exosomal integrin αvβ5 was liver-tropic (117). The uptake of exosomal integrin by local cells activates Src phosphorylation and pro-inflammatory factors to allow PMN establishment (117).

In gastric cancer liver metastasis, TDEs deliver membrane EGFR to liver stromal cells prior to liver metastasis (118). Recipient cells then activate hepatocyte growth factor paracrine, which provides a favorable environment for tumor landing by binding to their c-MET receptor (118). The lymphotropic gastric cancer cells produce CD97-enriched TDEs, which effectively aid gastric cancer metastasis by creating distant lymphatic PMN (119). Additionally, PMCs are peritoneum-specific cells and, therefore, the PMC-targeting exosomes mentioned above are considered peritoneum-tropic (101–103). Before gastric cancer peritoneal metastasis, Wnt3a-containing TDEs induce PMC infiltration into the gastric wall to create PMN in these sites, which in turn promoted subserosal invasions of gastric cancer cells and further dissemination (120). In addition, three miRNAs, namely miR-10b-5p, miR-101-3p, and miR-143-5p, are proposed biomarkers for gastric cancer lymph node, ovarian, and liver metastasis, respectively (121).

## Implications and Perspectives

### Biomarkers and Liquid Biopsy

The lipid bilayer membrane structure of the exosome maintains the stability of its cargo well, so exosomes have great potential as non-invasive biopsy specimens for cancer detection and prognosis (122, 123). So far, we may see potential roles of exosomes as biomarkers in gastric cancer metastasis prediction, prevention, and treatment. Some studies have shown evidence of clinical relevance between exosome and gastric cancer metastasis (summarized in **Table 1**). For example, exosomal PSMA3 and PSMA6 are explicitly enriched in serum during metastatic gastric cancer, but not primary gastric cancer, thus being a potential biomarker for gastric cancer metastasis (124). Further, exosomal miR-10b-5p, miR-101-3p, and miR-143-5p were proposed biomarkers for gastric cancer lymph node, ovarian, and liver metastasis, respectively, helping distinguish gastric cancer patients with various types of metastasis (121). Exosomal miRNAs from peritoneum lavage fluid, including miR-21 and miR-1225-5p, are specifically elevated in gastric cancer peritoneal metastasis after curative gastric cancer resection, thus providing a novel approach to early diagnosis of peritoneal dissemination of gastric cancer (125). However, evidence is limited up to date.

**Table 1:** Evidence of clinical relevance between exosomal contents and metastatic sites in gastric cancer.

Exosomal contents	Origin	Associated metastatic sites	Citation
miR-10b-5p	Plasma	Lymph node	(121)
miR-101-3p	Plasma	Ovary	(121)
miR-143-5p	Plasma	Liver	(121)
miR-21	Peritoneum lavage fluid	Peritoneum	(124)
miR-320c			
miR-1225-5p			
let-7g-5p	Serum	Nerve infiltration	(123)
miR-146b-5p			
PSMA3	Serum	Distant metastasis	(125)
PSMA6			
EGFR	Serum	Liver	(118)
MT1-MMP mRNA	Serum	Lymph node	(93)

### Cancer Prevention and Treatment

The characteristics of exosomes, including high biocompatibility, safety, and nano-sized diameters, allow effective drug-loading capacity and long blood circulation half-life. Thus, they serve as an ideal system to deliver cytokines, DNA, RNA, adjuvants, and even vaccines for treatment (126, 127). For example, HGF siRNA packed in exosomes can be transported into gastric cancer cells, where it decreases tumor growth rates and blood vessels *in vivo* (128). Exosomes from heat-treated gastric cancer malignant ascites have improved immunogenicity, being able to promote dendritic cell maturation and induce a tumor-specific cytotoxic T cell response (66). The organotropic factors, such as integrins on the exosome surface, could be used to improve targeting specificity and deliver drugs to specific tissues (129). However, it remains unknown whether exosome-based drug delivery could be used to prevent or treat gastric cancer metastasis to a specific organ.

### Perspectives

Despite immature clinical usage of exosomes in cancer, we believe it will be an important utility in metastasis assessment, prevention, and treatment in the future. First, methods such as nano-plasmonic sensors (14), microfluidic exosome analysis (130), and surface plasmon resonance imaging (131) were developed for exosome analysis with a small amount of sample, compared with routine methods. These techniques will allow exosomes to be a good tool for liquid biopsy. Second, because cancer cells, immune cells, and stromal cells all produce exosomes into the circulation, they will provide comprehensive information from tumor environments and PMN, as long as we have effective ways of exosome classification (132). An interesting technique utilizes a proximity barcoding method to profile surface proteins on individual exosomes, which allows both tissue origination and quantification of exosomes in mixed samples (133). Further classification based on exosome function, organophilicity, biological distribution, and immunogenicity, at single exosome levels, may be considered (134). Third, besides exosome-based drug delivery, another direction is to target PMN-promoting exosomes, based on their essential role in coordinating PMN formation. Although not feasible currently, targeting exosomes may come true in the future with fine characterizations of specific cell-derived exosomes and membrane markers for targeting. Fourth, some chemotherapeutics can induce certain exosome secretions and alters exosome composition, which degrades ECM and activates macrophage to release TNF-α (135). Therefore, whether these “chemo-exosomes” are PMN-promoting may be taken into consideration when choosing therapeutic regimens. Last but not least, despite of insufficient evidence, some studies have reported how oncogenes, cytokines, and exosomes interacted with each other in cancer, as reported in a squamous cancer cell line that cortactin promotes exosome secretion (136). Targeting genes or cytokines that regulate exosome packaging and secretion would be another way for exosome elimination and PMN inhibition.

## Conclusion

Exosomes play a vital role in establishing PMN formation by immunosuppression, angiogenesis, stroma remodeling, PMC MMT, and organotropism and have great potential in metastasis prediction, prevention, and treatment. Yet, their clinical usage is limited, and further studies are needed to validate the translational value of exosomes in PMNs of gastric cancer.

## Author Contributions

JG searched databases and collected studies. JG and SL summarized the contents, wrote the manuscript, and drew the figures. QX, XZ, MH, and XD helped in data collection and figure design. LL designed this review, edited the article, and supervised the work. All authors contributed to the article and approved the submitted version.

## Funding

This work was supported by the National Natural Science Foundation of China (81172487 to LL and 81500092 to SL) and Natural Science Foundation of Shandong Province (ZR201702180008 to LL).

## Conflict of Interest

The authors declare that the research was conducted in the absence of any commercial or financial relationships that could be construed as a potential conflict of interest.
